# Supplementary material for: Effects of Psychotropic Medication on Somatic Sterol Biosynthesis of Adult Mice
Source: Biomolecules. 2022 Oct 21;12(10):1535. doi: 10.3390/biom12101535 (PMC9599595; doi:10.3390/biom12101535)
Supplement: Supplementary file 1 [file biomolecules-12-01535-s001.zip › biomolecules-1947922-supplementary.pdf]

biomolecules

MDPI

Supplementary Material

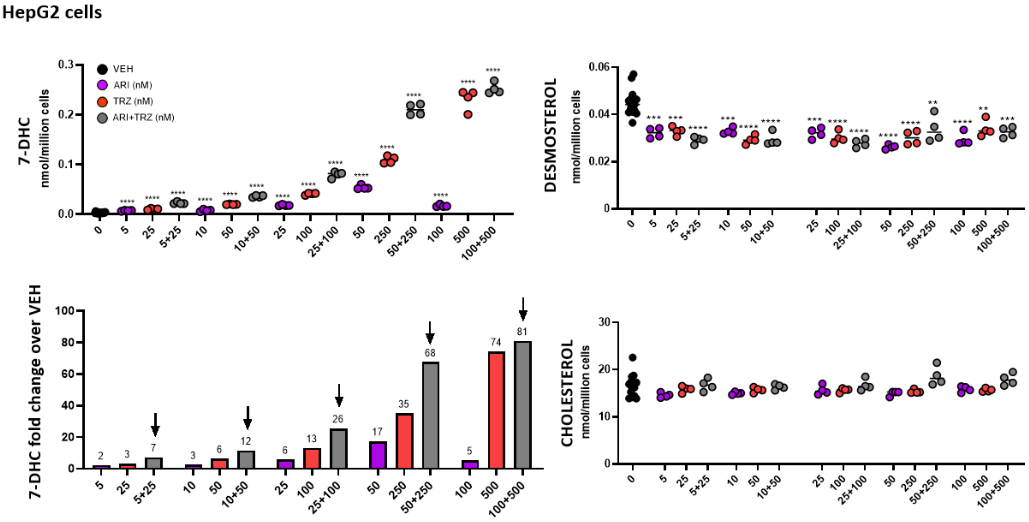

Figure S1. Dose-dependent effects ARI, TRZ and ARI + TRZ on sterol levels in HepG2 cells. HepG2 cells were cultured for 48 h, and treated with five different concentrations of ARI, TRZ or ARI + TRZ. Significance is denoted with black asterisks, **p < 0.01, ***p < 0.001, ****p < 0.0001. Note that ARI and TRZ polypharmacy treatment elevated 7-DHC levels in a synergistic fashion up to the highest concentrations used (black arrows). All three treatments (ARI, TRZ and ARI + TRZ) resulted in a significant decrease of DES in comparison to vehicle-treated cells.

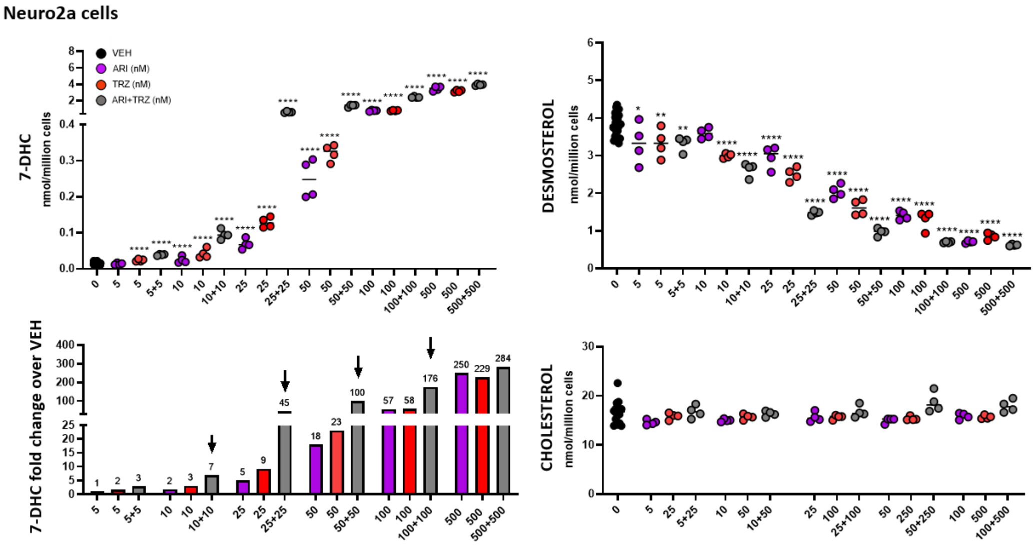

Figure S2. Dose-dependent effects ARI, TRZ and ARI + TRZ on sterol levels in Neuro2a cells. Neuro2a cells were cultured for 48h, and treated with six different concentrations of ARI, TRZ or ARI + TRZ. Significance is denoted with black asterisks, *p < 0.05, **p < 0.01, ****p < 0.0001. Note that ARI and TRZ polypharmacy treatment elevated 7-DHC levels in a synergistic fashion in the mid-concentration range (black arrows). All three treatments (ARI, TRZ and ARI + TRZ) resulted in a significant decrease of DES in comparison to vehicle-treated cells.

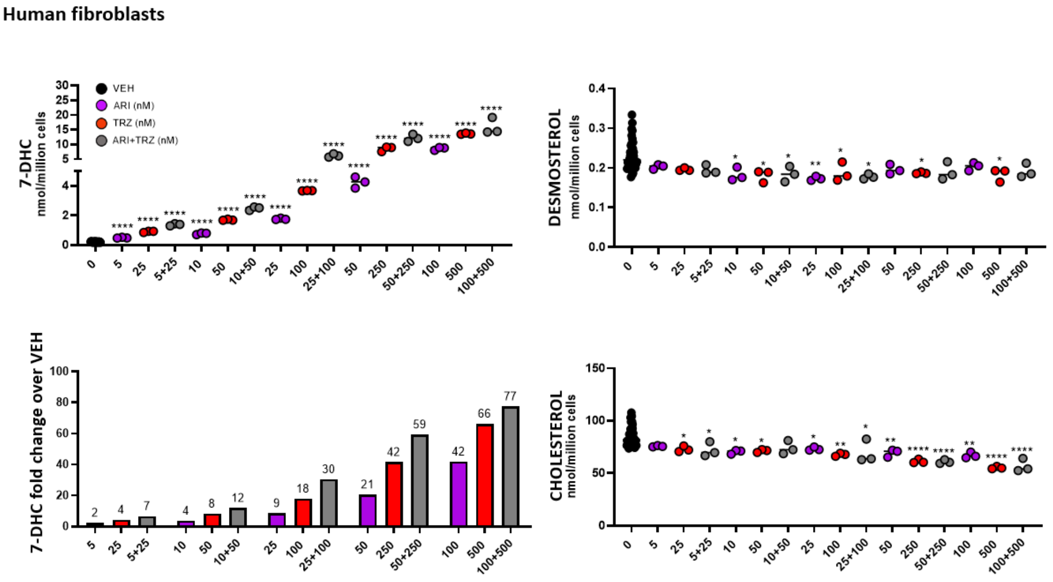

Figure S3. Dose-dependent effects ARI, TRZ and ARI + TRZ on sterol levels in human dermal fibroblasts. Human fibroblasts were cultured for 7 days, and treated with five different concentrations of ARI, TRZ or ARI+TRZ. Significance is denoted with black asterisks, *p < 0.05, **p < 0.01, ****p < 0.0001. Note that ARI and TRZ polypharmacy treatment elevated 7-DHC levels in a summative fashion across all concentrations. The treatments (ARI, TRZ and ARI + TRZ) resulted in a small decrease in DES and CHOL levels in comparison to vehicle-treated cells.

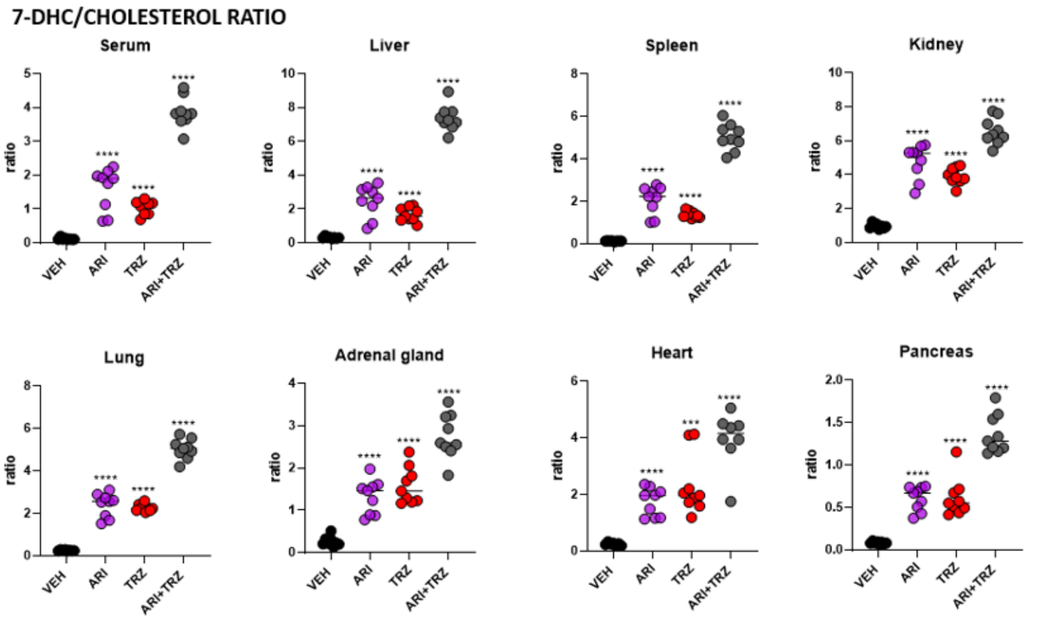

Figure S4. 7-DHC/CHOL ratio across serum and peripheral organs of TRZ, ARI and TRZ + ARI treated mice. Note the strong significant increase in 7-DHC/CHOL ratio in all three experimental groups, with the largest effect observed with ARI + TRZ treatment. Significance is denoted with black asterisks, ***p < 0.001, ****p < 0.0001.

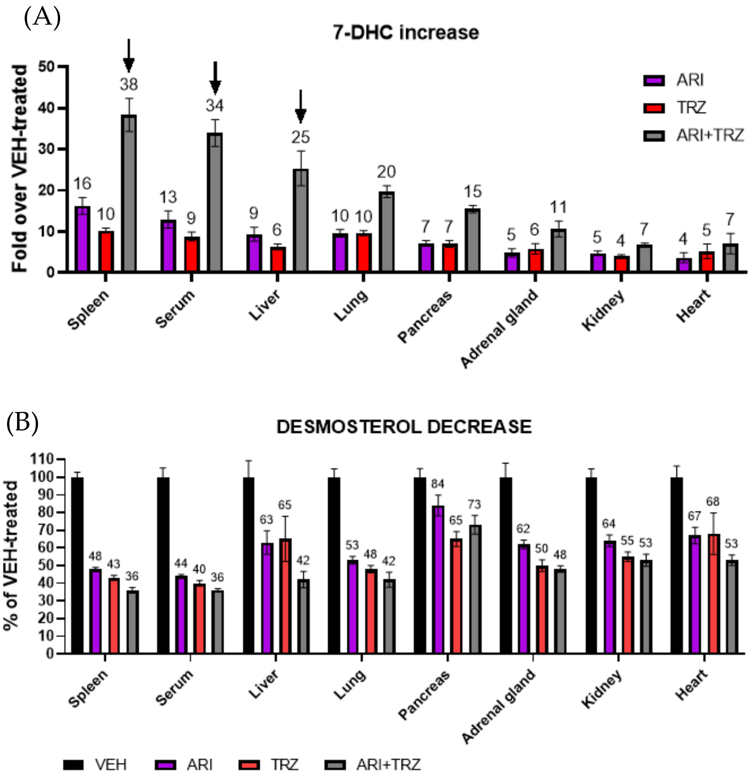

Figure S5. 7-DHC and DES changes across the mice serum and organs as a result of ARI, TRZ and ARI+TRZ treatment. (**A**) Serum and organs are represented on X axis, Y axis represents average fold change of 7-DHC increase over vehicle treated control samples. Numbers above columns represent mean groupwise fold change over vehicle treated control animals. (**B**) Y axis denotes percentage of DES level in comparison to vehicle-treated controls (100%). Numbers above columns represent the percentage of residual DES levels upon treatment. Error bars represents SEM in both panels.
